# The Clinical Challenge of Refractory Octreotide-Induced Thrombocytopenia in Active Gastrointestinal Bleeding: A Case Report

**DOI:** 10.7759/cureus.34590

**Published:** 2023-02-03

**Authors:** Mohammad Abu-Abaa, Omar Jumaah, Sindhu Chadalawada, Salman Kananeh, Manish Gugnani

**Affiliations:** 1 Internal Medicine, Capital Health Regional Medical Center, Trenton, USA; 2 Pulmonary and Critical Care Medicine, Capital Health Regional Medical Center, Trenton, USA

**Keywords:** immune thrombocytopenia purpura, thrombocytopenia, liver cirrhosis, gi bleeding, esophageal varices, octreotide

## Abstract

The association between octreotide and thrombocytopenia has been documented in the literature but it remains a rare finding. We are reporting a 59-year-old female patient with alcoholic liver cirrhosis who presented with the gastrointestinal tract (GIT) bleeding secondary to esophageal varices. Initial management involved fluid and blood products resuscitation and initiation of both octreotide and pantoprazole infusion. However, the abrupt onset of severe thrombocytopenia was evident within a few hours of admission. Platelet transfusion and discontinuation of pantoprazole infusion failed to correct the abnormality prompting the holding off of octreotide. However, this also failed to control the decline in platelet count and prompted intravenous immunoglobulin (IVIG). This case helps to remind clinicians to closely monitor platelet count once octreotide is initiated. This allows early detection of the rare entity of octreotide-induced thrombocytopenia, which can be life-threatening with extremely low platelet count nadir.

## Introduction

In the setting of the active gastrointestinal tract (GIT) bleeding secondary to esophageal varices, medical management of variceal bleeding involves vasoactive agents, blood transfusion, and antibiotics in combination with band ligation. Octreotide as well as proton pump inhibitors (PPI) are the mainstay of management of esophageal variceal bleeding in patients with portal hypertension. A new onset worsening thrombocytopenia in this setting is quite challenging clinically. Both octreotide and PPI have been associated with immunological thrombocytopenia. Octreotide is a somatostatin analog that works by inducing vasoconstriction and reducing variceal blood flow. It prevents rebleeding but does not improve mortality [1]. Common side effects include gallstones and hyperglycemia [1]. Octreotide-induced thrombocytopenia is an extremely rare adverse effect. There are only a few case reports of this entity [2-5].

## Case presentation

A 59-year-old female patient presented to the emergency department (ED) with three days of nausea and vomiting and two episodes of hematemesis and melena. Her past medical history is remarkable for alcohol use disorder, cirrhosis, and chronic pancreatitis. In ED, vital signs included a temperature of 36.9 degrees Celsius, heart rate of 133 beats per minute with sinus tachycardia rhythm, respiratory rate of 24 cycles per minute, blood pressure of 60/40 mmHg, and SpO_2_ of 90% on room air. Physical examination showed lethargy but full orientation with conjunctival pallor, scleral icterus, signs of dehydration, and soft mild to the moderately distended non-tender abdomen and normoactive bowel sounds. No evidence of cutaneous bleeding was seen. Basic lab work showed a drop in hemoglobin to 7.5 g/dL from a baseline of 12.1 g/dL eight months prior, normal mean corpuscular volume (MCV) of 91 fL, elevated red blood cell (RBC) distribution width coefficient of variation (RDW-CV) 23.8% (reference 11.6-15.4%), platelet 224,000 cells/MCL, white blood cell (WBC) 20,000 cells/mL, prothrombin time (PT) 20 seconds, partial thromboplastin time (PTT) 39 seconds, international normalized ratio (INR) 1.6, and elevated lactic acid 13.4 mmol/L. She also had elevated total bilirubin 9.7 mg/dL, alkaline phosphatase 138 U/L, aspartate transaminase (AST) 717 U/L, and alanine transaminase (ALT) 83 U/L with elevated creatinine 2.2 mg/dL from a normal baseline renal function. Potassium, sodium, and magnesium levels were 4.3 mmol/L, 134 mmol/L, and 1.9 mg/dL respectively. Model for End-Stage Liver Disease Sodium (MELDNa) score was elevated at 22 with Maddrey's discriminant function 28.1. She failed to respond to fluid bolus and required initiation of norepinephrine infusion. Pantoprazole and octreotide infusions were also initiated. Also, she was started on a massive blood transfusion and received 6 units of packed RBC and 4 units of fresh frozen plasma.

Norepinephrine infusion was tapered off gradually over the next several hours and hemoglobin improved to 11.4 g/dL. However, the platelet count dropped to 130,000 within 6 hours of admission and then 11,000 cells/MCL within the first 12 hours of admission requiring platelet transfusion. This has only temporarily improved her platelet to 27,000 cells/MCL then dropped again to 7,000 cells/MCL within 36 hours of admission requiring a second platelet unit. Both pantoprazole and octreotide were suspected as offending agents. The second platelet transfusion did not improve with discontinuation of pantoprazole infusion and immune thrombocytopenic purpura secondary to octreotide was suspected and the infusion was discontinued after undergoing esophagogastroduodenoscopy (EGD) showed grade 3 esophageal varices and underwent banding. PT, PTT, and INR remained normal to slightly elevated throughout the stay and fibrinogen level was within normal limit and no schistocytes were seen on peripheral blood film.

However, despite the discontinuation of octreotide infusion and the second platelet unit, platelet count improved only to 33,000 cells/mL and dropped to 21,000 cells/mL within hours. Given active bleeding from the GIT, this prompted the initiation of intravenous immunoglobulin (IVIG). This led to an improvement in platelet count to 52,000 cells/mL within 24 hours. Pantoprazole infusion was restarted and normalization of platelet count above 150,000 cells/mL was achieved on day 5 after initiation of IVIG and she received four doses in total.

The bleeding was challenging to control and required a repeat EGD that revealed esophageal ulcers with stigmata of recent bleeding that required injection therapy. Ultimately, the bleeding ceased allowing the patient to be discharged. Platelet count remained stable off of octreotide (see Table 1).

**Table 1:** Lab Findings A tabular format of lab findings during the period of hospitalization. MCV: mean corpuscular volume, WBC: white blood cells, RDW-CV: red blood cell width distribution coefficient of variation, PT: prothrombin time, PTT: partial thromboplastin time, INR: international normalized ratio, AST: aspartate transaminase, ALT: alanine transaminase

Variable	Baseline 8 Months Prior to Admission	On Admission	12 hours after admission	24 hours after admission	36 hours after admission	48 hours after admission	50 hours after admission	Five days later	On Discharge	Reference Range
WBC	8,800	20,180	13,810	16,000	14,000	15,400	13,600	13,600	12,000	4,000-10,000 cells/mL
Hemoglobin	11.6	11.4	7.5	10.1	9.5	9.2	8.4	8.1	8	11.2-15.7 g/dL
MCV	101.5	107.5	91.8	95.6	93.1	98	95.8	110.4	97.9	79-95 fL
RDW-CV	17.6	23.8	17.7	21.1	19.2	21.6	21	22.7	24.4	11.6-15.4%
Platelet	199,000	224,000	11,000	27,000	7,000	33,000	21,000	152,000	164,000	150,000-400,000 cells/mL
PT	14.9	20.1	18	-	-	-	18.9	22.2	22.2	12.4-14.8 seconds
PTT	32	39	34	-	-	-	36	35	36	24-36 seconds
INR	1.1	1.6	1.4	-	-	-	1.5	1.9	1.9	0.8-1.1
Total Bilirubin	1.8	9.7	6.7	7.5	7.2	6.8	7.6	10.7	18.3	0.2-1.3 mg/dL
AST	73	717	546	553	504	329	179	112	95	14-36 U/L
ALT	62	83	76	71	67	59	45	37	38	0-34 U/L
Creatinine	0.6	2.2	1.12	1.16	1.09	0.86	0.94	0.98	0.84	0.52-1.04 mg/dL

## Discussion

The true incidence of octreotide-induced thrombocytopenia is uncertain given the rarity of this adverse effect. However, several cases were reported and common findings among these cases included an abrupt decline in platelet count with different times to nadir and gradual improvement in platelet count after discontinuation of octreotide [2-5]. A review of these reports shows that the lowest nadir in platelet count is 28.000 cells/mL. Days to nadir ranged from 2 to 10 days and days to recovery of platelet count ranged from two to nine days. In contrast, the nadir in our patient was even lower at 7.000 cells/mL and the nadir was reached within 36 hours after octreotide initiation.

On the other hand, PPI-induced thrombocytopenia is also an extremely rare entity [6,7]. One retrospective study of 35 patients suggested an association between pantoprazole and thrombocytopenia [8]. However, a larger study involving 468 patients failed to replicate this finding [9]. There is no estimated incidence and hence it remains a diagnosis of exclusion.

The immunological mechanism is likely responsible for both octreotide and pantoprazole-induced thrombocytopenia and involves the formation of antibodies directed against surface glycoproteins on platelets [10]. The sensitizing drug binds to both the antibody and glycoprotein epitopes on the platelet surface, which induces the immunological destruction of platelets. These antibodies may be derived from a pool of naturally occurring immunoglobulins and are weakly reactive. These antibodies are specific only to the sensitizing drug and usually, thrombocytopenia becomes evident within one to two weeks of the exposure. However, acute drug-induced thrombocytopenia has been reported in association with antithrombotic agents including tirofiban, abciximab, and eptifibatide. In this case, thrombocytopenia becomes evident within seconds to minutes [11]. Another theory is based on the direct drug toxic effect on hematopoietic cells [12]. Another suggested mechanism is bone marrow suppression, and a limitation of our report is the lack of bone marrow biopsy [13].

The lack of an established methodology for causality assessment prompted the development of the Naranjo scoring system. Naranjo scoring questionnaire is frequently used to evaluate the causal relationship between a medication and an adverse effect [14]. It has 10 questions and a total score determines the strength of the causal relationship with established improvement in reliability, reproducibility, and inter-rater agreement. Its strength is that it remains valid even if some questions cannot be answered. The calculated Naranjo score of our patient with octreotide-induced thrombocytopenia is five based on one point for the presence of prior conclusive reports, two points for the temporal relationship between starting octreotide and onset of thrombocytopenia, one point for lack of thrombocytopenia when re-challenged with pantoprazole, and one point for recovery after octreotide discontinuation. Naranjo scores between five to eight indicate a probable causal relationship.

The average time of recovery from drug-induced thrombocytopenia is around one week [15]. Most of the reported cases showed spontaneous recovery of platelet count after octreotide discontinuation [2-5]. However, in our patient, discontinuation of octreotide and platelet transfusion was not enough to hold the decline in platelet count and prompted the initiation of IVIG in the setting of active bleeding.

## Conclusions

New onset thrombocytopenia in active GIT bleeding secondary to esophageal varices should include PPI and octreotide-induced thrombocytopenia in the differential diagnosis. Discontinuation of octreotide usually allows recovery. However, refractory thrombocytopenia may require IVIG. This case helps to remind clinicians to remain vigilant about the possibility of octreotide-induced thrombocytopenia in those who are being treated for variceal bleeding. This requires close monitoring of platelet count as nadir platelet count can be life-threatening. This allows for earlier detection and prompt discontinuation of octreotide.
